# TDCS to the right anterior temporal lobe facilitates insight problem-solving

**DOI:** 10.1038/s41598-020-57724-1

**Published:** 2020-01-22

**Authors:** Carola Salvi, Mark Beeman, Marom Bikson, Richard McKinley, Jordan Grafman

**Affiliations:** 1 Department of Psychiatry, University of Texas at Austin, Austin, TX USA; 2 Department of Psychology, Northwestern University, Chicago, IL USA; 3 Department of Biomedical Engineering, The City College of New York, New York, USA; 4 Air Force Research Laboratory, Wright-Patterson AFB, Dayton, OH USA; 5 Shirley Ryan AbilityLab, Chicago, IL USA; 6 Departments of Physical Medicine and Rehabilitation, Neurology, Cognitive Neurology, and Alzheimer’s Center, Department of Psychiatry, Feinberg School of Medicine, Northwestern University, Chicago, IL USA

**Keywords:** Human behaviour, Neurology

## Abstract

Problem-solving is essential for advances in cultural, social, and scientific knowledge. It is also one of the most challenging cognitive processes to facilitate. Some problem-solving is deliberate, but frequently people solve problems with a sudden insight, also known as a Eureka or “Aha!” moment. The advantage of solving problems via insight is that these solutions are more accurate, relying on a unique pattern of neural activity, compared to deliberative strategies. The right Anterior Temporal Lobe (rATL), putatively involved in semantic integration, is distinctively activated when people experience an insight. The rATL may contribute to the recognition of distant semantic relations that support insight solutions, although fMRI and EEG evidence for its involvement is, by nature, correlational. In this study, we investigate if focal sub-threshold neuromodulation to the rATL facilitates insight problem-solving. In three different groups, using a within- and between-subjects design, we tested the causal role of this brain region in problem-solving, by applying High Definition Transcranial Direct Current Stimulation to the rATL (active and sham condition) or the left frontopolar region while participants attempted to solve Compound Remote Associates problems before, during and after stimulation. Participants solved a higher percentage of problems, overall, and specifically by insight when they received rATL stimulation, compared to pre-stimulation, and compared to sham and left frontopolar stimulation. These results confirm the crucial role played by the rATL in insight problem-solving.

## Introduction

Have you ever pondered a difficult problem when you suddenly became aware of the correct solution, with surprise and confidence – an Aha! experience? Such solutions are reached with sudden insight, and they have the advantage of being highly accurate^1–8^. A similar Aha! experience can also occur when you decipher an ambiguous perception, comprehend a joke, or suddenly grasp a metaphor^9^.

Recent behavioral, electrophysiological and neuroimaging techniques have identified patterns of neural activity, networks, and biomarkers characterizing insight problem-solving that differ from the neural activity induced when people solve the same problems more deliberatively^10–19^. Based upon this first wave of studies, and thanks to the introduction of new techniques of neurostimulation, identifying the causal relationship of brain regions to insight problem-solving is now achievable when studying healthy humans.

When people attempt to solve Compound Remote Associate (CRA) problems, they recognize solutions more quickly when the solution-related information is presented in the *left* visual hemifield, suggesting that information processes in the right hemisphere play an essential role in problem-solving. This is particularly true if they recognize solutions with an Aha!-like feeling of sudden insight^20,21^. Experiments using EEG or fMRI revealed increased neural activity (specifically gamma -γ band, −40 Hz for the EEG experiment) over the right Anterior Temporal Lobe (rATL) just before participants solved problems via insight, compared to when they reported solving problems via analytic processing^10,13,22,23^. Recently, Santarnecchi *et al*.^24^, used transcranial Alternating Current Stimulation (tACS) to induce α (10 Hz) and γ (40 Hz) activity in the right parietal and temporal lobes, respectively, during healthy volunteer performance on two problem-solving tasks. The authors found that only tACS at 40 Hz over the rATL improved the accuracy of problems solved via insight. Although these results were significant only for the CRA task, they suggest the possibility of enhancing insight problem-solving by applying noninvasive brain stimulation over the rATL.

Several brain regions involved in attention and cognitive control differentiate insight from deliberate analytic problem-solving^12–14^. RATL activity is thought to underlie the semantic integration of *distantly* related associations that contribute to language comprehension^25^ and to creative problem-solving^17,26^, a characteristic that makes this brain area a potential key region for facilitating insight if stimulated. Other studies support the idea that right temporal semantic processing appears to contribute to understanding metaphors^27^, is involved in the development of distant semantic relations, humor and other forms of implicit comprehension^28–31^.

Despite the key role assigned to the rATL in facilitating the processing of distant semantic associate relationships, and specifically insight problem-solving^22,24^, only a few studies have attempted to investigate the causal role of this brain network node using transcranial Direct Current Stimulation (tDCS)^32–35^. TDCS applies a constant, low current (1–2 mA) to the brain area of interest via electrodes on the scalp. TDCS has been shown to increase neuronal excitability of the cortex underneath the anode for as long as 90 minutes post-stimulation, modulating cognitive functions associated with the excited cortical structures^36–38^. The behavioral effects can last even longer (e.g.^37^). Among the studies using tDCS, Chi and Snyder found increased problem solutions using conventional tDCS targeting the right, but not the left, anterior temporal lobes when participants tried to solve two kinds of ‘classic insight problems’: matchstick problems (27 trials in the 2011 study) or the nine dots problem (in the 2012 study)^33,39^ using a between-subject design. Specifically, in the 2011 study they tested 60 participants divided in 3 groups, assigned to three types of conventional tDCS (at 1.6 mA for a maximum of 17 minutes): cathodal stimulation of the left ATL together with anodal stimulation of the right ATL; anodal stimulation of the left ATL together with cathodal stimulation of the right ATL; and a sham group. In the 2012 study, the same authors found that 42.4% of their participants (14 out of 33 total) solved the nine-dot problem when receiving cathodal stimulation of the left anterior temporal lobe together with anodal stimulation of the right anterior temporal lobe.

Even if these studies seemed promising, their replication in a within-subjects’ design, with an increased number of problems, failed^32^. Specifically, this latter includes 66 participants tested twice (under-stimulation and sham condition) using 19 matchstick problems and 20 remote associates per session. The stimulation lasted 25 minutes and was set at 1.6 mA. Unfortunately, the results of these studies do not allow us to draw strong conclusions on the effects of tDCS over the rATL on insight problem-solving. All of these experiments indeed lack focality of stimulation since they used conventional tDCS with relatively large sponge pads (7 × 5 cm in^32^), which elicit a diffuse rather than focused current flow. Thus, an experimental design involving a large sample size, more trials, and a more focal current flow is needed to draw generalizable conclusions.

Historically, insight problem-solving was only investigated by administrating a type of problem (the so-called ‘classic insight problems’) commonly used to study insight under the assumption that ‘insight problems’ induced only insight solutions^26^. However, it remains possible that on some or many trials, people solve ‘insight problems’ via analytic processes; thus, determining whether people solved with insight may be better indicated by requiring participants to report how they solved the problem on each trial (e.g.^40,41^). Critically, reliable and valid information regarding the problem-solving style adopted on each trial (via insight or via analysis) is needed.

**Figure 1:**
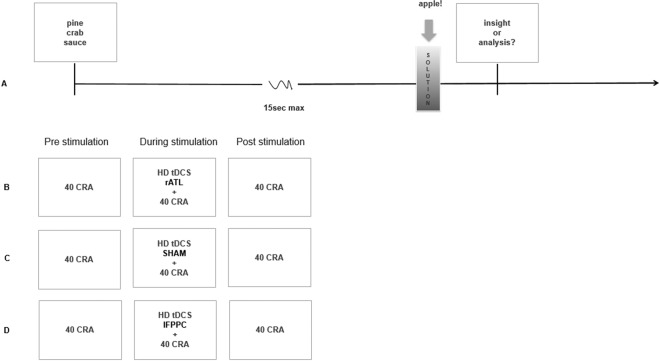
(**A**) Each CRA problem consisted of the simultaneous presentation of three words, each of which could form a compound word or phrase with the solution word [e.g., for pine/crab/sauce - the solution word is APPLE]. If participants solved the problem, they reported the solution word to the experimenter. At the beginning of the experiment participants were trained to identify whether they used an analytic or insight strategy when they successfully solved a problem, so at the end of each trial, after saying the solution word, they could report how they solved the problem, either via insight or via analysis, by clicking one of two buttons on the mouse. The following instructions were given to participants to explain how to distinguish a solution via insight from one via analysis: “*You will decide whether the solution was reached with insight or with analysis. With INSIGHT means you experienced a so-called Aha! moment and the solution came to mind as a sudden surprise. It won’t be a huge Eureka, just a small surprise and it may be difficult to articulate how you reached the solution. With ANALYSIS it means that you reached the solution gradually, part by part. You might have used a deliberate strategy or just trial-and-error and you can report the steps you used to solve the problem. We know it is not always obvious whether you used insight or analysis, and you may feel as though you used a mixture of both. But we need you to choose one, so please choose whichever method your problem-solving process most closely resembles. No solution type is better or worse than the other; there are no right or wrong answers in reporting insight or analysis.”* Participants were divided in three groups (**B**–**D**). Every participant attempted to solve a first block of 40 trials (pre-stimulation baseline condition), after which the HD tDCS to the rATL (**B**), sham (**C**) or to the lFPC (**D**) was applied and participants attempted to solve the second block of 40 trials. At the end of 20 minutes of stimulation, the HD tDCS participants were administered the post-stimulation session.

In the study reported here, we used a CRA task with a large set of 120 problems; recruited a large sample of participants, used an experimental between- (two stimulation groups and one sham group) and within-subjects design (the 120 problems were divided into three blocks of 40 trials administered pre, during and after stimulation); and we instructed participants to report whether they used an analytic or insight solution strategy (see Fig. 1 for the structure of each trial and experimental design).

Further, improvements in the delivery of tDCS may clarify the reported effects. Prior studies used conventional tDCS arrays, with the cathode electrode placed far from the anode (often over the homologous contralateral site), producing a diffuse current through the brain, likely influencing both cortical and deep structures^42–44^ and leaving localization of the current flow difficult to ascertain. As evidenced by combined tDCS/fMRI studies^45,46^, this diffuse pattern of current flow makes it difficult to establish causality between modulated activity at the target site and the corresponding behavioral changes^47–49^. In the current study, we used a targeted high definition tDCS (HD tDCS) montage at 2 mA intensity. This new electrode arrangement has demonstrated its efficacy for inducing neurophysiological changes and neuroplasticity in the primary motor cortex^50,51^ and enhancing creative skills^52,53^.

We reasoned that other brain regions on the left hemisphere, involved in semantic integration and cognitive control, might also differentiate insight from deliberate analytic problem-solving allowing us to draw stronger conclusions on the specialization of semantic integration of the rATL in insight^52–54^. Therefore, we used the left frontopolar cortex (lFPC) as a second stimulation site. This brain area appears involved when jointly considering, or integrating, several distinct ideas or the outcomes of two or more separate cognitive operations^55–57^. Imaging studies on creativity reported left frontopolar activity varying with increasing semantic distance during an analogical reasoning task^58,59^. Green and colleagues observed that changes in connectivity and activity over the frontopolar cortex are associated with the conscious enhancement of performance on a creative relational cognition task^58^. LFPC has also been seen involved when people perform step-by-step reasoning (i.e., when they have to keep in mind a main goal while performing concurrent sub-goals), a process generally required in problem-solving, planning and reasoning^60^. Correlational experiments using single-photon emission tomography found an increased level of regional cerebral blood flow in the left pre-frontal cortex during the performance on the Tower of London task (a well-known problem that requires analytical trial-and-error solving)^61^.

Thus, while the lFPC has not been found to be involved in insight problem-solving, it appears to have a critical role in creative ideation and problem-solving in general. Therefore, we hypothesized that HD tDCS over the lFPC could either enhance problem-solving overall, especially solutions using an analytic strategy, but not solutions via insight.

## Methods

### Subjects

One-hundred-and-twenty participants were randomly assigned to three groups receiving active (group 1, section B in Fig. 1) HD tDCS over the rATL, sham (group 2, section C in Fig. 1) HD tDCS, or active HD tDCS over the lFPC (group 3, section D in Fig. 1). A total of 11 participants were excluded for: poor tDCS contact quality (4 participants); reported solving all the problems via insight/analysis (2 participants); solved less than 10% of the problems (2 participants); at the end of the experiment revealed they were not 100% native speakers (2 participants); misunderstood the instructions (1 participant). Thus, our analyses were run on three groups of N = 36 (23 females; rATL group); N = 36; (23 females; sham) and N = 37 (25 females; lFPC group).

Only participants that fulfilled the following criteria were considered suitable and recruited for the study: (1) right-handed; (2) Native English speakers; (3) no history of neurological or psychiatric disorder; (4) no use of central nervous system or mood affecting drugs (such as antidepressants or anxiety medications); and (5) no history of intracranial metal implantation. The study was approved by the institutional review board of Northwestern University. Although subjects were informed about the procedures of this study, they were not aware of our hypotheses until they were debriefed at the end of each session.

### Sample size estimation

A statistical power analysis was performed for sample size estimation, based on data from our previous studies investigating insight problem-solving using CRAs^12,62–64^. A priori power analysis was conducted to test the difference in a within- between-subjects interaction at an effect size of 0.25, and an alpha of 0.05. The result showed that a total sample of 36 participants per group was enough to achieve a power of 0.80.

The sub-groups of 36 and 37 participants were also comparable with, or greater than, group sizes used in recent studies that have detected effects of tDCS on creativity^54,65–67^.

### Task

Participants attempted to solved 120 CRAs^68^ divided into 3 blocks of 40 trials each. CRAs were divided into 3 lists, controlled for problem difficulty, randomly distributed in the three blocks that were presented pre-, during- and post-stimulation. (see normative data^68^ for problem difficulty). Participants were instructed to identify whether they used an analytic or insight strategy when they solved a problem. (see Fig. 1 for instructions). Six practice trials were performed to ensure participants learned to distinguish between insight and analysis. Self-reports differentiating between insight and analytic problem- solving are reliable and associated with behavioral and neuroimaging markers^12,13,23,68^. Each problem consisted of the simultaneous presentation of three words, each of which could form a compound word or phrase with the solution word. For the full list of the problems and solution rates, see^68^. The CRA words were presented in black font on a white background in normal horizontal orientation above, at, and below the center of the monitor (Fig. 1). The experimental procedure was presented using E-Prime 2.10 on a 24-in Dell screen at a viewing distance of about 60 cm.

CRAs have been shown to correlate with several measures of creative performance e.g., the Raven’s Standard Progressive Matrices, classic insight problems, the Alternative Uses Task and the Creative Achievements Questionnaire^69^.

### Neuromodulation

**Figure 2:**
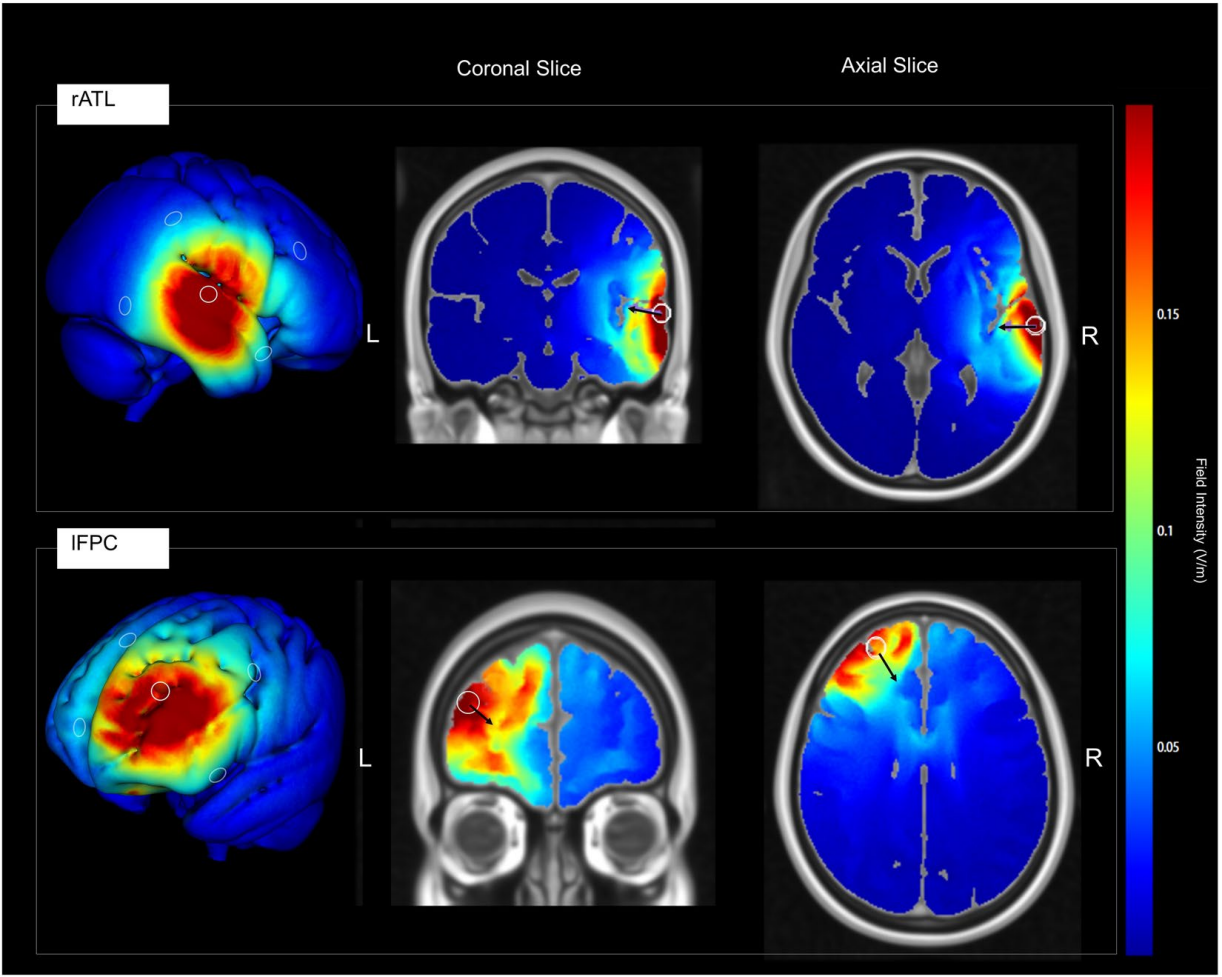
HD-tDCS electrode montage and electrical field models for rATL (top panel) and lFPC (bottom panel) stimulation sites. The electrical field modeling was performed in HD-Explore 3.01 (Soterix Medical, Inc.) using the MNI-152 template. The white circles represent the anode electrode location (where the field intensity was maximized). Black arrows depict the directional current flow vectors.

We used a High Definition version of tDCS (HD-tDCS) which targets specific brain regions by restricting the current flow to the circumscribed ring^42,70^. This new electrode arrangement has demonstrated its efficacy for inducing neurophysiological changes and neuroplasticity in the primary motor cortex^50,51^ and enhancing creative skills^52^. Electrode placement for the HD-tDCS protocol used the EasyCap (EasyCap, Herrsching, Germany) electrode cap, modified according to the standard EEG MCN system landmarks. Our stimulation procedure was derived using the Soterix 4 × 1 Multichannel Stimulation Interface (Soterix Medical, Inc., New York, NY) at 2 mA for 20 minutes, which previous studies have used to improve creative performance e.g.^52^. The electrode montage was designed with HD-Explore Version 3.1a software (Soterix Medical, Inc.) using the MNI-152 template, to optimize current flow to the rATL. HD-Explore uses finite element models to estimate the electric field induced by a given HD-tDCS electrode montage. For groups that received right temporal stimulation, the anode was placed at the 10–10 electrode site T8, and cathodes were placed at sites FC6, FT10, TP10, and CP6 (Fig. 2 for details). This configuration provided the optimal trade-off of focality and magnitude of the modeled electrical field in the rATL at the site previously reported to result in peak activity during insight problem-solving^23^. The tDCS was applied through ring electrodes, which were placed in plastic electrode holders fitted into the EasyCap and filled with electro-conductive gel (Signa gel). A current of 2 mA was applied at the anodes for 20 minutes. In the sham condition, tDCS was applied using a 30-s ramp up followed 15 s later by a 30-s ramp down of activity. Half of the participants received the sham stimulation over the rATL, the remaining over the lFPT.

For the lFPT group, the anode was placed at 10–10 electrode site AF3, and cathodes were placed at sites FPz, Fz, F7, and FC3. This same configuration was adopted by Green and colleagues^52^, and it is considered to provide the optimal balance of focality and magnitude of the modeled electrical field in lFPC at the location of peak activity as reported in a recent paper on augmenting state creativity^58^, while minimizing outgoing, potentially inhibitory, current flow through the left medial prefrontal cortex.

### Procedure

Participants received verbal and written instructions before starting the experimental procedure on how to judge each solution process as insight *versus* analytic and a six trials practice after which they assured the experimenter they understood the task. Each trial started with a 1 s fixation cross followed by the presentation of a READY prompt and a second 1 s fixation cross. Afterward, the three CRAs’ problem words were presented on the screen. Participants had 15 s to solve the problem and they were instructed to press the space bar as soon as they attained the solution. Following their verbally reported solution, or the end of the time limit (15 s), the problem words were erased and, if solved, participants had to report how they solved the problem: via insight or analysis. Participants were instructed that there was no optimal problem-solving style nor right or wrong answers. The following trial started right after participants reported how they solved the problem. No feedback was given if the participant ran out of time or provided the wrong solution. At the end of the experiment, participants were paid $40 for their time.

The procedure was identical for each block of 40 trials, the only difference was whether active or sham HD TDCS was applied during the second block. Participants attempted to solve the first block of 40 trials (pre-stimulation baseline condition), after which the HD tDCS (or sham stimulation) was applied and participants attempted to solve the second block of 40 trials. At the beginning of the stimulation, we assessed the level of stress and comfort on a scale of 1 to 10 each. Participants were informed that “*they would have gotten used to the stimulation whiten 2*–3 *minutes*”. The CRAs appeared only when participants felt comfortable, and ensured the experimenter that the stimulation would have not distracted them from solving problems (on average after 4 minutes). (The specific instructions were: “*We are going to be stimulating for twenty minutes, and at the beginning of stimulation, the tDCS will slowly ramp-up to full intensity. During that time, it feels different for every person. Some people feel it as a kind of heating sensation. Some people feel it as pinpricks. Still, no matter what you feel right at that first peak intensity level, the sensation will taper off quickly after the first couple of minutes. So, we are going to make sure that you’re absolutely comfortable with it before we start with the stimulation. Are you ready to start?*).

When participants ended the block of 40 problems earlier than 20 minutes the experimenter waited for the end of the stimulation time in silence. In this case, participants were instructed to wait until the end of the stimulation in front of a black screen. Participants were banned from using any device or technology during that time.

Following 20 minutes of stimulation, participants were administered the post-stimulation session of 40 trials. This condition allowed us to examine the longevity of any lasting effect. No breaks were taken between blocks. Two experimenters were involved in the study: Experimenter 1 was blind to the sham or stimulation group (B, C, and D in Fig. 1), instructed the participants, and recorded the accuracy score. In all of the stimulation/sham condition Experimenter 2 was the only one knowing if the stimulation was active or sham. Experimenter 2 entered the room following the pre-stimulation block and set the HD tDCS to the stimulation or sham condition without the knowledge of Experimenter 1 and left the room before the post-stimulation session began.

## Results

Behavioral data analysis was performed using SPSS 22.0 and the significance level was set to a *p* < 0.05. Data were tested for normality (Kolmogorov–Smirnov test) and homogeneity of variance (Levene’s test). Data were normally distributed and assumptions for the use of analysis of variance were not violated. Only problems correctly solved were used for the analyses.

Participants in the pre-stimulation condition solved an average of 36.5% (SD 13.8%) of the problems given (across the 3 groups). Participants in the sham condition solved overall an average of 36.4% (SD 11.8%) of the problems given. The solution rate for these baseline conditions is similar to the rates reported in past studies (e.g.^4,9,63^). Overall the rATL stimulation group solved 43.2% (SD 12%) of the problems, whereas the lFPC stimulation group solved 39.1% (SD 12%) of the problems. (Percentages are calculated based upon the total number of CRA problems administered and averaged across participants). Participants’ average percent for each condition are reported in Table 1.

**Table 1:** Averages of percent of problems solved correctly per participants, across the three groups, in pre-, during, and post-stimulation conditions, overall and solved via insight *vs*. via analysis.

	rATL		Sham		rPFC	
	Average percent	SD	Average percent	SD	Average percent	SD
Problems solved	43.2	12.0	36.4	11.8	39.1	11.9
Pre-stimulation	38.1	14.9	36.1	14.4	35.3	12.3
Insight	23.5	8.8	21.0	8.0	20.0	13.4
Analysis	15.3	10.6	15.7	9.0	18.4	12.5
During stimulation	45.6	11.7	36.9	13.0	39.6	11.8
Insight	28.3	10.7	21.5	9.6	20.9	12.9
Analysis	17.7	9.8	15.8	12.3	19.4	12.0
Post-stimulation	46.0	13.1	36.9	11.7	37.9	13.2
Insight	28.2	13.4	21.7	10.1	18.5	12.8
Analysis	18.3	11.5	14.9	8.3	21.5	14.9

We first compared the percent of overall problems solved within the pre-, during, and post-stimulation conditions between the three groups.

Participants in the rATL stimulation group solved a significantly higher percent of problems during and after- stimulation compared to participants in the sham group and participants in the lFPC group. A 3 (rATL stimulation *vs*. sham *vs*. lFPC stimulation) x 3 (pre- *vs*. during- *vs*. post-) repeated measures ANOVA showed a significant between-subjects effect F(2, 107) = 3.64; *p* = 0.03; η_p_^2^ = 0.064; a significant interaction effect of groups*time of stimulation (F(4, 214) = 3.23; *p* = 0.01; η_p_^2^ = 0.057; and a main effect of time (F(2, 214) = 10.22; *p* < 0.001; η_p_^2^ = 0.087.

Post-hoc tests of 18 comparisons [(1) pre- *vs*. during within the rATL group, (2) pre- *vs*. post- within the rATL group, (3) during *vs*. post- within the rATL group; (4) pre- *vs*. during within sham group, (5) pre- *vs*. post- within sham group, (6) during *vs*. post- within sham group; (7) pre- *vs*. during within lFPC group, (8) pre- *vs*. pos-t within lFPC group, (9) during *vs*. post- within lFPC group. Within the pre-stimulation condition (comparisons 10 to 12), during the stimulation (comparisons 13 to 15), and post-stimulation (comparisons 16 to 18)] were conducted, using a Bonferroni-adjusted alpha level of 0.05/18 = 0.0028. Results show a significant difference between: pre- and during rATL stimulation *t* (35) = −4.32; *p* = 0.00012, *d* = −0.41; pre- and post rATL stimulation *t* (35) = −4.56; *p* = 0.00008, *d* = −0.43.

In a different analysis, we investigated if this result was driven by a specific problem-solving style (insight *vs*. analysis). A 3 (rATL *vs*. sham *vs*. lFPC) x 3 (pre -*vs*. during *vs*. post-) 2 (insight *vs*. analysis) ANOVA shows a significant interaction effect of solving style*stimulation group (F(2, 107) = 3.15; *p* = 0.015; η_p_^2^ = 0.004); a significant interaction effect of time*stimulation group (F(2, 107) = 3.50; *p* = 0.034; η_p_^2^ = 0.027); a main effect of time (F(2, 214) = 6.53; *p* = 0.002; η_p_^2^ = 0.004); and a main effect of problem solving style (F(1, 107) = 12.22; *p* < 0.001; η_p_^2^ = 0.048). Bonferroni post hoc of comparisons [(1) pre- *vs*. during, insight within the rATL group, (2) pre- *vs*. post-, insight within the rATL group, (3) during *vs*. post, insight within the rATL group, (4) insight *vs*. analysis, pre-stimulation within the rATL group, (5) insight *vs*. analysis, during stimulation within the rATL; (6) insight *vs*. analysis, post-stimulation within the rATL. Within the pre-stimulation condition via insight (comparisons 7 to 8), wgitin the during stimulation condition via insight (comparisons 9 to 10), and within the post-stimulation condition via insight (comparisons 11 to 12). Within the pre-stimulation condition via analysis (comparisons 13 to 14), within the during stimulation condition via analysis (comparisons 15 to 16), and within the post-stimulation condition via analysis (comparisons 17 to 18)] were conducted at alpha level of 0.05 / 18 = 0.0028. Results show a significant difference between: pre- vs. during stimulation, insight within the rATL group *t* (35) = −4.10; *p* = 0.00023, *d* = *−*0.68; pre- vs. post- stimulation, insight within the rATL group *t* (35) = −2.76; *p* = 0.008, *d* = −0.44; insight vs. analysis, during stimulation within the rATL *t* (35) = −3.7; *p* = 0.00073, *d* = −0.62; insight vs. analysis, post- stimulation within the rATL *t* (35) = −2.84; *p* = 0.0007, *d* = *−*0.47.

No significant difference was detected in the analysis of problems solved incorrectly. No significant difference was detected in the sham condition between the montage applied over the rATL and the lFTP.

In addition to the percent of problems solved we ran the same analysis using reaction times. The only significant difference found was between problems solved via insight during rATL stimulation (they were faster) and post- rATL stimulation *t* (35) = −2.085; *p* = 0.04, *d* = −0.34).

In sum, these results show that HD tDCS over the rATL increases the percent of problems solved overall and specifically via insight when compared to a sham condition and stimulation of the lFPC supporting the causal involvement of the rATL in insight problem-solving. Results were significant in both the within- and between-subjects’ comparisons as participants solved more problems via insight during HD tDCS over the rATL compared to baseline (in the within-subject analysis) and compared to the sham condition (in the between-subject design). These effects lasted through the post-stimulation condition (i.e., 20 minutes).

## Discussion

In this study, we evaluated whether the rATL has a causal role in problem-solving by testing the effects of HD tDCS over the rATL and the lFPC in problem-solving using a between- and within-subjects experimental design. Results show that when participants received HD tDCS over the rATL, they improved problem-solving overall and specifically solutions via insight, compared to both sham and lFPC, and this improvement lasts for at least 20 minutes after stimulation. These results, not only converge with previous fMRI and EEG studies that showed greater spontaneous activity in the rATL during insight problem-solving (e.g.^23^; for a review see^17^), but they also establish this brain area as a key region for facilitating insight. We believe that this insight solution effect is due to the rATL’s role in semantic integration, necessary to achieve global coherence during reasoning and discourse processing^25^. Following this idea, other studies indicate that right hemisphere semantic processing contributes to understanding novel metaphoric expressions, implicit comprehension, and humor^28–31,71^.

Conversely, the left hemisphere supports finer semantic coding, with more targeted neural activity leading to one, or a few, dominant interpretations or alternative meanings^72–74^. As we noted above, the left hemisphere also plays a role in problem-solving and creativity, however, there is an open debate on the role of the left frontal cortex in the field of creativity. Therefore, we chose an active control engaging a brain region that is implicated in functions important for integrative processes in creativity and problem-solving to gain information on its role in insight. Cerruti and Schlaug^65^ first, and later Zmigrod, Colzato and Hommel^35^, were able to improve CRA participants’ performance by applying anodal tDCS to the left dorsolateral prefrontal cortex (DLPFC) when compared to cathodal or sham stimulation of the same region, as well as compared to anodal stimulation over the contralateral right DLPFC. However, Metuki, Sela, and Lavidor^75^ did not replicate these findings, yet they enhanced solution recognition during anodal stimulation over the left prefrontal cortex only for more difficult CRA problems. A recent study using 7 T fMRI, reports finding the cortical activation of the left anterior middle temporal gyrus and the DLPFC (plus robust subcortical activity changes) when people solve Remote Associates via insight^76^. Again, these researchers, in another study, showed that participants who received cathodal tDCS over the lDLPFC were more likely to solve matchstick problems when they require relaxation of previously learned constraints, compared to participants who received anodal or sham tDCS^77^. The authors explained the results by attributing to the left DLPFC a role in the relaxation of learned constraints, leading to a successful representational change and therefore solutions via insight^78^.

Based on fMRI results supporting the hypothesis that generating creative uses of an object reduces needs for filtering of low-level object properties (e.g., the shape or materials of the objects)^78^ Chrysikou *et al*.^54^, showed that cathodal stimulation over the left DLPFC is associated with better performance in the Alternative Uses Tasks, which benefits from unfiltered, bottom-up information, but not when asking to participants to come up with common uses for objects, which benefits from prefrontal cortex top-down regulation. Overall, this evidence would suggest that certain tasks might benefit from a temporary disengagement of PFC regulatory mechanisms. These studies are driven by the idea that demands of cognitive flexibility benefit from a state of lower cognitive control and reflect a lack of information filtering.

In this study, we chose the Green and colleagues’ stimulation site and model given that they enhanced creative ideation when applying HD tDCS over the lFPC during an analogical reasoning task^52^ and data on the possible causal involvement on this brain area in insight/analytical problem-solving were missing. In our study using we find a difference in the percent of problems solved via analysis on the post-stimulation lFPC condition which did not turn out significant using Bonferroni correction for alpha level of 0.0028 (the results were lFPC *vs*. sham analysis, post-stimulation *t* (36) = −2.59; *p* = 0.014, *d* = *−*0.42). We acknowledge that this difference was not significant, and it was only in the post-stimulation condition and only when the average percent of problems solved was compared to the sham condition. That said, whe believe that more studies are needed to further explore this potential effect to firmly establish the role of lPFC in problem-solving via analysis.

We also acknowledge that the time each participant took to solve problems varied the time spent in silence waiting for the end of the stimulation within a range of few minutes maximum (participants had a maximum of 15 s to solve each problem, and they had to press the button as soon as they found the solution). Although this might represent a limitation of the study that we could not control (since it depended upon participants’ individual variability in solution times) we believe this factor was controlled by having a control site stimulation group.

To conclude, this study directly compared the causal role of rATL and lFPC regions in different problem-solving styles. Using HD tDCS we identified a causal relationship between active rATL stimulation and increased insight problem-solving. Consistent with the recent results obtained by Santarnecchi and colleagues^24^, our results indicate that inducing insight can be achieved using non-invasive brain stimulation to the rATL. Our study design considered several flaws in previous studies using non-invasive brain stimulation during insight problem-solving, by including increasing statistical power and by having two control conditions. We also used HD tDCS that promotes a more focal current flow. We acknowledge that since problem-solving can vary by context, problem type, and the solution used, it is likely that the rATL and the lFPC are only two nodes in a distributed network in the brain contributing to problem-solving. Other brain areas besides the rATL and the lFPC may be preferentially involved in solving other types of problems via insight or solving similar problems when insight solutions are not required. For example, Santarnecchi and colleagues’ results were significant only for CRA problems but not for Rebus puzzles^24^. Thus, future research needs to address if the tDCS effect is limited only to CRA problems or can be extended to a different class of problems (i.e., Rebus puzzles).

Our results provide novel evidence that tDCS can enhance creative performance and idea generation in problem-solving. We believe that achieving this knowledge will open up to new avenues of research on how to improve creativity, for example, in contests of education, rehabilitation and at work^79^.
